# Spatio-ecological determinants of *Biomphalaria* and *Bulinus* snail intermediate hosts and schistosome-like infections in the Lango subregion, Northern Uganda: a geostatistical approach to guide targeted disease control

**DOI:** 10.1186/s13071-026-07610-y

**Published:** 2026-08-18

**Authors:** John Paul Byagamy, Robert Opiro, Margaret Nyafwono, Harriet Angwech, Geoffrey Maxwell Malinga, Richard Echodu, Emmanuel Igwaro Odongo-Aginya

**Affiliations:** 1 Department of Environment and Natural Resources Management, Faculty of Agriculture and Environment, Gulu University, Gulu, Uganda; 2 Department of Biology, Faculty of Science, Gulu University, Gulu, Uganda; 3 Department of Microbiology and Immunology, Faculty of Medicine, Gulu University, Gulu, Uganda; 4 Department of Health Services, Lira District Local Government, Lira, Uganda

**Keywords:** *Biomphalaria*, *Bulinus*, Freshwater snails, Geostatistics, Hotspot mapping, Lango subregion, Schistosomiasis, Spatial analysis, Uganda

## Abstract

**Background:**

Freshwater snails of the genus *Biomphalaria* transmit *Schistosoma mansoni* (intestinal schistosomiasis), *Bulinus* species transmit *S. haematobium* (urogenital schistosomiasis), and *Lymnaea* species transmit *Fasciola* spp. (fascioliasis). These diseases are of major public health and veterinary concern in sub-Saharan Africa. In Uganda's Lango subregion, schistosomiasis remains endemic despite control efforts, yet comprehensive spatial and ecological analyses of snail intermediate host distributions are lacking. This study employed geostatistical approaches to identify high-density snail habitats and sites where schistosome cercarial shedding was detected (as a proxy for potential infection, without molecular confirmation) to inform further surveillance and consideration of targeted control strategies.

**Methods:**

A cross-sectional study was conducted during the dry and rainy seasons of 2023 across 26 georeferenced sites in Lira and Kole districts. Freshwater snails were collected using standardized methods and identified morphologically using Brown (1994) and Mandahi-Barth (1988) keys. Cercarial shedding tests determined patent infection status but could not definitely distinguish human-infective from non-human schistosome species without molecular confirmation. Physicochemical parameters (pH, salinity, total dissolved solids, dissolved oxygen, temperature, and conductivity) and ecological variables were measured. Spatial analysis included Moran’s I for autocorrelation, Getis-Ord Gi* for hotspot detection, and inverse distance weighting for interpolation. Generalized linear mixed models with spatial random effects were used to assess predictors of snail prevalence and density, compared with non-spatial models using the Akaike Information Criterion. Dry and rainy season data were treated as separate observations from the same sites (*n* = 52 site-season observations), with site included as random effect to account for repeated measures.

**Results:**

A total of 4802 snails from 13 species were collected, with *Biomphalaria choanomphala* (25.8%, *n* = 1241) being most abundant. *Bulinus africanus* (17.7%, *n* = 852) and *Lymnaea natalensis* (10.6%, *n* = 510) were also abundant. Significant spatial clustering was detected for *B. choanomphala* (Moran’s *I* = 0.32, *p* = 0.004) and *B. sudanica* (Moran’s *I* = 0.24, *p* = 0.018). Three density hotspots and sites where cercarial shedding was detected (descriptive only given the small number of infected snails) were identified, primarily in rice paddies and swamps near human settlements. Overall patent infection rate (based on cercarial shedding) was 0.15% (5/3404 tested snails). Species-specific rates were *B. choanomphala* 0.16% (2/1,241), *B. sudanica* 0.13% (1/749), and *B. africanus* 0.23% (2/852). No schistosome-like infections were detected in *Lymnaea* snails or other genera. Spatial GLMMs outperformed non-spatial models (ΔAIC = 12.7–15.3), revealing significant effects of salinity (odds ratio = 0.21, *p* < 0.001), total dissolved solids (*β* = − 0.03, *p* = 0.002), dissolved oxygen (*β* = 0.54, *p* = 0.003), and anthropogenic activities. Spatial random effects accounted for 18–24% of residual variation.

**Conclusions:**

This study demonstrates the added value of geostatistical methods in identifying snail intermediate host clusters and sites with detected schistosome-like infections. The integration of spatial analysis with ecological modeling provides a robust framework to guide further surveillance and consideration of targeted snail control interventions. Our findings suggest that focused surveillance in identified high-density areas, integration of spatial risk maps into district health planning, and community engagement in modifying high-risk water contact sites could be considered to help reduce schistosomiasis transmission in the Lango subregion. These suggestions require validation through longitudinal studies before implementation at scale.

**Graphical Abstract:**

**Figure:**
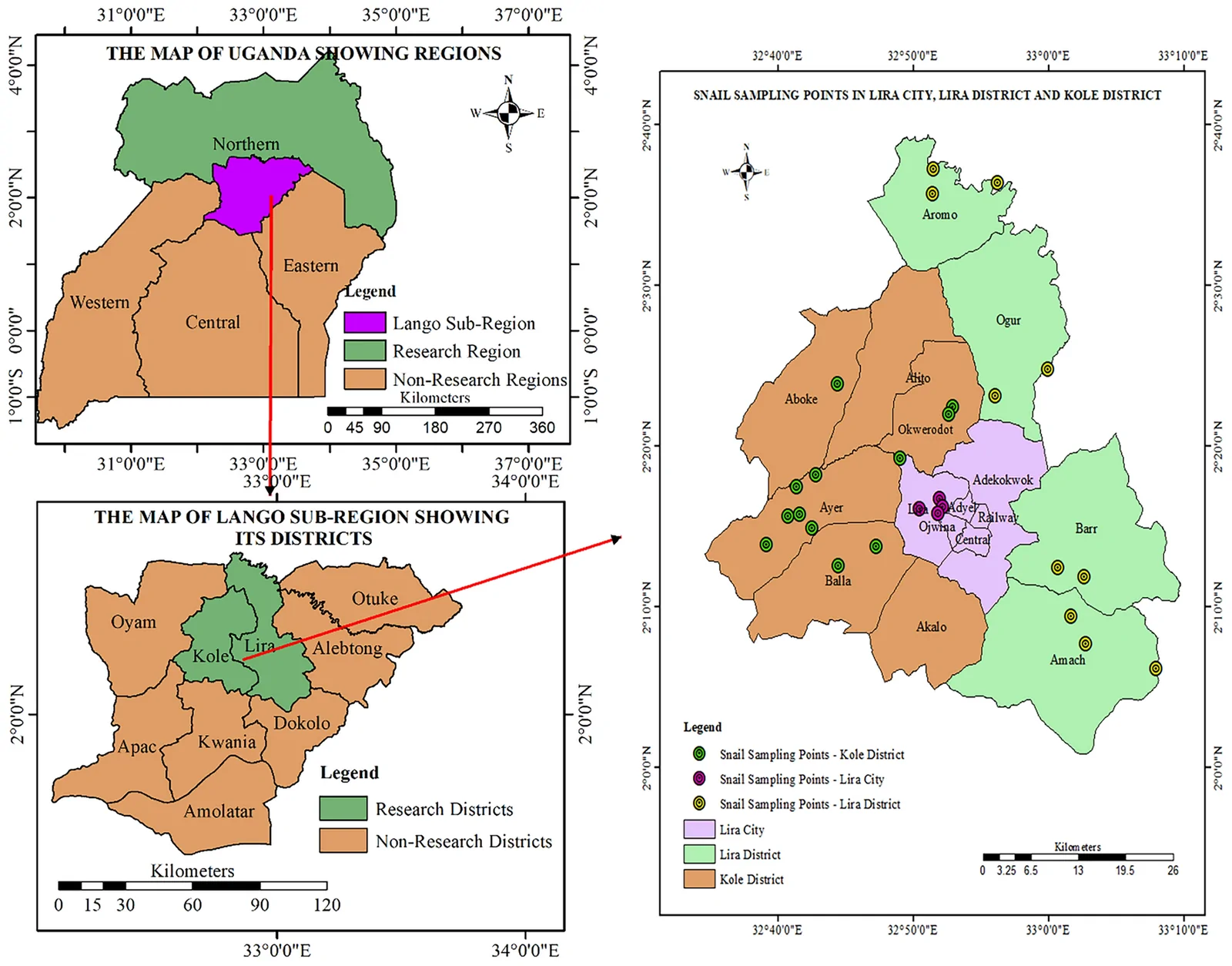

**Supplementary Information:**

The online version contains supplementary material available at https://doi.org/10.1186/s13071-026-07610-y.

## Background

Freshwater snails of the class Gastropoda play crucial ecological roles in aquatic ecosystems but also serve as intermediate hosts for medically important trematodes, particularly those causing schistosomiasis and fascioliasis [1]. Globally, schistosomiasis affects approximately 250 million people, with over 90% of cases occurring in sub-Saharan Africa [2, 3]. In Uganda, schistosomiasis is endemic in approximately 81 districts, with an estimated 5.4 million people infected and 14.7 million at risk [4, 5]. The disease burden is particularly high among school-aged children in endemic areas, leading to anemia, impaired growth, and reduced cognitive development [6, 7].

In the Lango subregion, where livestock keeping, particularly cattle, is a central livelihood activity, fascioliasis poses additional economic burdens through reduced milk production, liver condemnation at slaughter, and decreased animal productivity. This dual public health and veterinary relevance justifies the inclusion of *Lymnaea* snails in our investigation [8, 9]

The transmission dynamics of snail-borne diseases are closely connected to the distribution and abundance of specific freshwater snail intermediate hosts. *Biomphalaria* species transmit *Schistosoma mansoni* (intestinal schistosomiasis), *Bulinus* species transmit *S. haematobium* (urogenital schistosomiasis), and *Lymnaea* species transmit *Fasciola* spp. (fascioliasis) [8, 9]. These snails thrive in various freshwater habitats, including streams, ponds, swamps, and irrigation schemes, with their distribution affected by complex interactions between physicochemical factors, ecological conditions, and human activities [10, 11].

It should be noted that while *Schistosoma mansoni* is well documented as endemic in the Lango subregion, *S. haematobium* has historically been reported at lower prevalence or absent in this area. The inclusion of *Bulinus* species in this study acknowledges their potential role as intermediate host for both *S. haematobium* and other trematodes of veterinary importance, even if human *S. haematobium* transmission may be limited in this specific setting [16]

Previous studies in Uganda have mainly focused on the Lake Victoria basin, where high snail densities and infection rates have been documented [12–14]. However, northern Uganda features distinct ecological and climatic conditions, characterized by seasonal rainfall patterns, extensive wetland systems, and different land use practices [15, 16]. The Lango subregion, with about 30% of its area covered by swamps, rivers, and wetlands, presents a unique transmission setting that remains understudied [17, 18]. Recent urbanization and agricultural expansion, especially rice cultivation, have altered aquatic habitats, potentially creating favorable conditions for snail proliferation [19, 20].

It is important to distinguish between traditional seasonal wetlands, which dry periodically and may require snail aestivation for population persistence, and permanent irrigation schemes (including rice paddies with year-round water retention), which provide stable habitats supporting continuous snail breeding. These two habitat types likely differ substantially in their spatio-ecological determinants, though our study design grouped them under the broader “swamp” and “rice paddy” categories [20, 21].

Ecological studies have identified various factors influencing snail distributions, including water temperature, pH, dissolved oxygen, conductivity, vegetation cover, and substrate type [21–23]. However, these factors often exhibit spatial heterogeneity, creating patchy distributions of snail populations and disease transmission risk [24, 25]. Traditional regression approaches that ignore spatial autocorrelation may produce biased estimates and inadequate predictions for targeted control [26, 27].

Geostatistical methods offer powerful tools for analyzing spatially referenced data in disease ecology. Spatial autocorrelation analysis identifies clustering patterns, hotspot detection pinpoints high-density areas, and spatial regression models account for geographic dependencies [28–30]. In schistosomiasis research, geostatistical approaches have been successfully applied in Kenya, Tanzania, and South Africa to map snail distributions and predict infection risk [31–33]. For instance, Manyangadze et al. [31] used Maxent modeling to identify suitable habitats for intermediate host snails in South Africa, while Nwoko et al. [34] employed spatial scan statistics to detect snail clusters in Nigeria.

Despite these advances, geostatistical analysis of snail intermediate host distributions remains rare in northern Uganda, specifically the Lango subregion. Most spatial studies have focused on the Lake Victoria basin [12–14], leaving a geographic knowledge gap regarding how snail populations are spatially structured in the distinct wetland systems of northern Uganda. This gap hampers the development of spatially targeted control strategies that could improve resource allocation and intervention success [35, 36]. The World Health Organization’s roadmap for neglected tropical diseases highlights the importance of targeted approaches based on local epidemiological and ecological data [37].

This study aimed to bridge this gap by (1) mapping the spatial distribution of freshwater snail intermediate hosts in the Lango subregion, (2) identifying clusters of high snail density and sites with schistosome-like cercarial shedding (as a proxy for infection, without molecular confirmation) using geostatistical methods, (3) assessing the combined influence of spatial, ecological, and physicochemical factors on snail prevalence and density, and (4) providing evidence-based considerations for spatially targeted snail control interventions. This study represents, to our knowledge, the first application of integrated geostatistical and spatial modeling techniques to map snail intermediate hosts and identify potential transmission sites in the understudied Lango subregion of Uganda.

The findings contribute to the growing body of literature on the spatial epidemiology of snail-borne diseases and support the development of integrated control strategies in northern Uganda.

## Methods

### Study area

This study was conducted in Lira and Kole districts within the Lango subregion of northern Uganda (2° 14′ 50.0″ N 32° 54′ 00.0″ E). The area experiences a tropical climate with bimodal rainfall: long rains from April to May and short rains from August to October, with dry seasons from November to March and June to August [38]. Mean annual rainfall ranges from 1200 to 1500 mm, and temperatures range from 25 to 30 °C [39]. The landscape is characterized by extensive wetland systems, rivers (including Aswa, Akore, and Obim), swamps (Okile, Okole, Onoo, Akalo, Ayer), and rice paddies that provide suitable habitats for freshwater snails [40]. The estimated population of the Lango subregion is 2.5 million, with increasing urbanization and agricultural intensification potentially impacting aquatic ecosystems [41].

### Study design and site selection

**Fig. 1:**
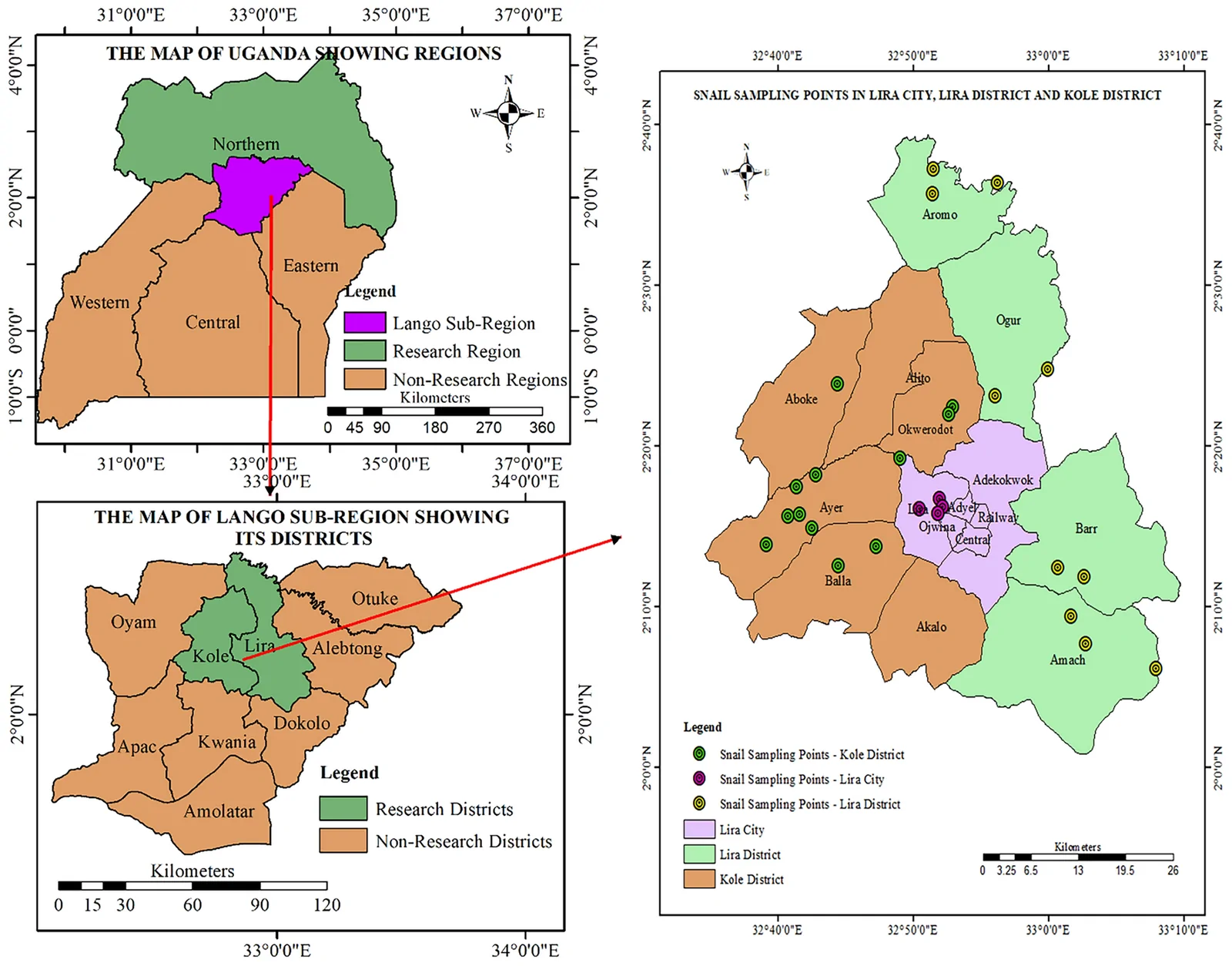
The study area and sampled sites in Lira city, Lira district, and Kole district, Lango subregion, northern Uganda

A cross-sectional study was conducted during the dry (January–April) and rainy (July–November) seasons of 2023. Twenty-six sampling sites (Fig. 1) were selected through stratified random sampling based on the following: (1) habitat type (streams, ponds, swamps, rice paddies, dams), (2) presence of human water contact activities, (3) spatial distribution across the study area, and (4) accessibility. Sites were georeferenced using a Garmin eTrex 30 × GPS receiver (accuracy ± 3 m). The sampling design ensured representation of different ecological zones and anthropogenic influences.

### Snail collection and processing

At each site, snails were collected for 30 min by two experienced malacologists following standardized protocols [42]. Visible snails were handpicked using forceps, and submerged vegetation and substrates were sampled using a 30 × 30 cm scoop net with 2 mm mesh (supplementary file 1). Collections were made along 10-m transects parallel to the shoreline, covering different microhabitats within each site. Snails were placed in labeled containers with site water and transported to the laboratory within 6 h. Site-level environmental and snail collection data are provided in supplementary file 3 (Table S1).

For density calculations, the sampled area per site was estimated as follows: transect length (10 m) × width of sampled strip (0.5 m, representing 0.25 m on each side of the transect line) = 5 m^2^ per site. This approach provides a standardized area denominator, though we acknowledge that effective sampling area may vary with habitat complexity and snail detectability. Therefore, density values are best interpreted as standardized relative abundance or catch-per-unit-effort (CPUE) rather than absolute population density [42].

**Fig. 2:**
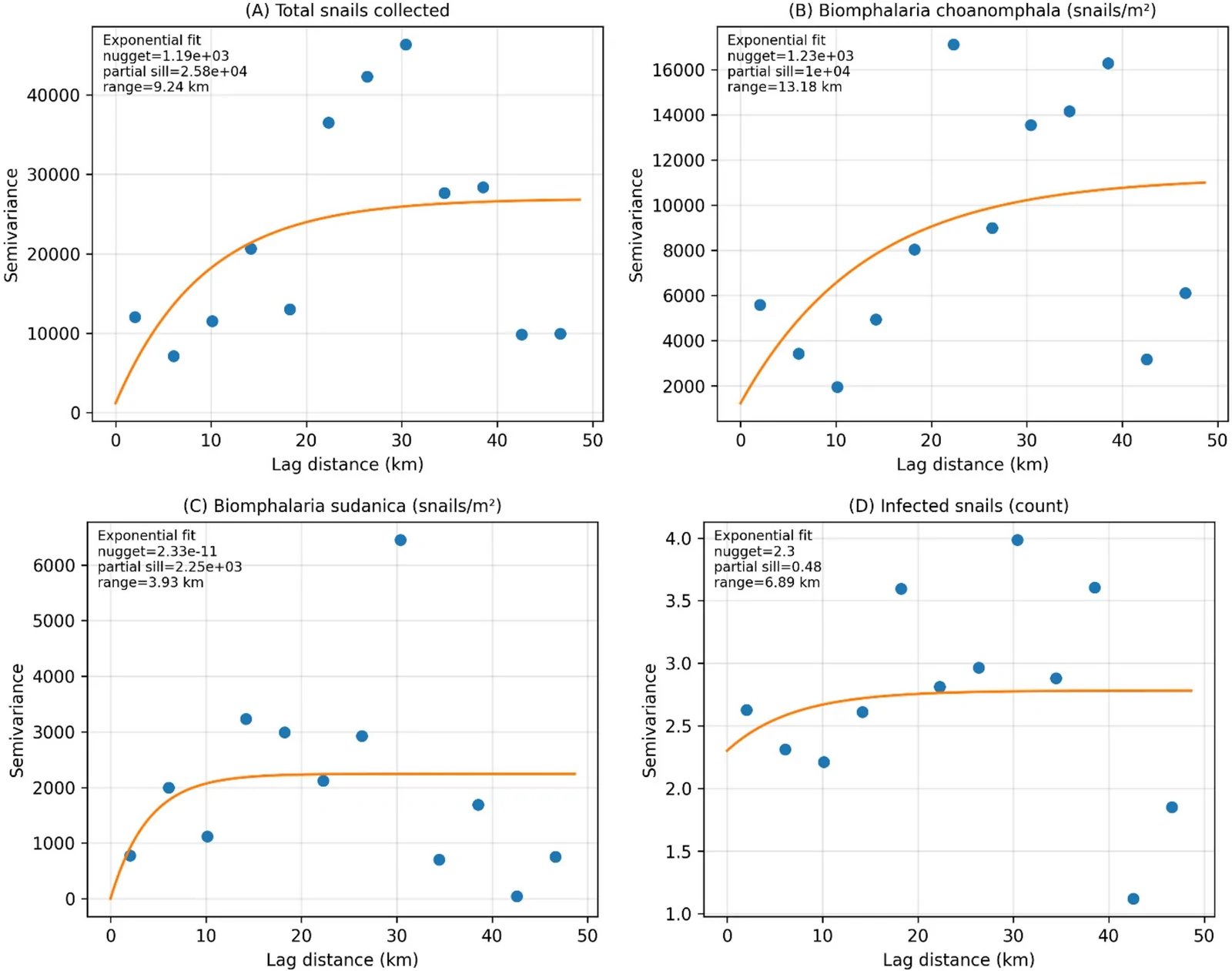
Variogram plots (exponential models) for the spatial analysis of freshwater snails and schistosome-like cercarial shedding

In the laboratory, snails were identified to species level using morphological characteristics based on Brown (1994) [1] and Mandahl-Barth (1988) [43], with species-level differentiation of *Biomphalaria* species relying on shell morphology, spiral angulation, and apertural characteristics as described in these keys. Voucher specimens were preserved in 70% ethanol for verification. Live snails of the genera *Biomphalaria* and *Bulinus* were screened for schistosome-like infection using cercarial shedding techniques [44]. Individual snails were placed in 12-well plates with 10 ml of filtered site water and exposed to artificial light (60-W bulb) placed 30 cm above the plates for 8 h (08:00–16:00) to induce cercarial shedding. This duration and timing were selected to capture peak shedding periods reported for both *Schistosoma* and *Fasciola* cercariae, though we acknowledge that optimal shedding timing may differ between trematode genera. Shed cercariae were examined under a dissecting microscope (Olympus SZ61) and identified morphologically [45, 46]. Snails shedding cercariae morphologically compatible with mammalian schistosomes were considered positive for patent infection (though molecular confirmation was not performed to distinguish between human-like and non-human schistosome species) (Fig. 2). This method detects patent infection but cannot definitely distinguish between human-infective and non-human schistosome species without molecular confirmation.

### Environmental and physicochemical measurements

At each sampling site, the following parameters were measured in triplicate between 8:00 and 11:00 AM:

Physicochemical parameters: Water temperature (°C), pH, salinity (g/L), total dissolved solids (TDS, ppm), conductivity (mS/cm), and dissolved oxygen (DO₂, mg/L) were measured using a calibrated multiparameter water quality meter (HI9829, Hanna Instruments, Sweden). The DO_2_ probe was calibrated daily using the Winkler titration method as a reference standard (APHA Method 4500-O). Values are reported as mg/L, with percentage saturation also recorded for quality assurance.

Hydrological characteristics: Water depth (m) was measured using a graduated pole at five points along each transect. Flow velocity (m/s) was estimated using float method in flowing waters. Water level was categorized as flooded, normal, or low based on seasonal observations.

Habitat characteristics: Substrate type (mud, sand, gravel, rock, concrete, peat), dominant vegetation (reeds, grasses, aquatic plants, water hyacinth, rice), and canopy cover (%) were recorded.

Anthropogenic factors: Human activities at each site were documented through direct observation and categorized as follows: washing/bathing, car/motorcycle washing, water collection, swimming/playing, rice cultivation, or fishing. The presence of domestic animals (cattle, pigs, goats) and wild animals (water birds, wild rats, carnivores) was noted.

Geographic data: GPS coordinates, altitude (m), and distance to nearest settlement (m) were recorded.

### Geostatistical analysis

#### Spatial autocorrelation

Global spatial autocorrelation in snail density and site-level prevalence/absence was assessed using Moran’s I statistic [47]. A row-standardized inverse distance weight matrix with a 2 km bandwidth was used based on estimated snail dispersal distances [48]. Significance was tested using 999 permutations, and variogram plots were used to characterize spatial dependence.

#### Hotspot analysis

Local clusters of high snail density (hotspots) and low density (coldspots) were identified using the Getis-Ord Gi* statistic [49]. Sites with Gi* > 1.96 (*p* < 0.05) were classified as hotspots, and those with Gi* < − 1.645 (*p* < 0.10) as coldspots.

#### Spatial interpolation

Continuous surfaces of snail density and key physicochemical parameters were generated using inverse distance weighting (IDW) interpolation [50]. IDW was selected over Kriging because (1) our primary interest was visualization of local patterns rather than prediction with uncertainty quantification, (2) the moderate sample size (*n* = 26) limits reliable variogram fitting required for Kriging, and (3) snail habitats are often patchily distributed, making IDWʹs assumption for local influence appropriate for exploratory mapping. However, we acknowledge that kriging would be preferable for predictive mapping with larger sample sizes.

### Statistical modeling

#### Model specification

Two types of models were developed and compared:

Non-spatial models: Generalized linear models (GLMs) with binomial distribution for prevalence (logit link) and negative binomial distribution for density (log link).

Spatial models: Generalized linear mixed models (GLMMs) incorporating Gaussian spatial random effects to account for autocorrelation [51].

For all models, dry and rainy season data from the same sites were treated as separate observations (*n* = 52 site-season observations: 26 sites × 2 seasons), with site included as a random effect to account for repeated measures. Prevalence was defined as site-season presence/absence of a given snail species. Density was defined as the number of snails collected per site-season divided by the sampled area (snails/m^2^), interpreted as standardized relative abundance.

#### Predictor variables

Initial candidate predictors included the following: physicochemical parameters (temperature, pH, salinity, TDS, conductivity, DO₂), hydrological factors (water depth, flow rate, water level), habitat characteristics (substrate, vegetation, canopy cover), anthropogenic factors (human activities, domestic/wild animals), geographic variables (altitude, distance to settlement), and season. A total of 16 candidate predictors were initially considered. Continuous variables were standardized (*z*-scores) for analysis.

#### Model selection and validation

Variable selection was performed using backward elimination based on Akaike Information Criterion (AIC) [52]. Given the modest sample size and the small number of positive observations, we prioritized model parsimony. Final models contained 4–6 predictors. We acknowledge that the logistic regression models for prevalence should be interpreted as exploratory given the limited number of positive observations (8 for *B. sudanica* and 11 for *B. choanomphala*). Multicollinearity was assessed using variance inflation factors (VIF < 5) [53]. VIF values for retained predictors ranged from 1.23 to 3.87. Salinity (VIF = 3.21), TDS (VIF = 3.45), and conductivity (VIF = 3.87) showed moderate correlation, as expected given these parameters are interrelated measures of water ionic content. We retained all three because they capture different aspects of water chemistry, but their coefficients should be interpreted collectively as indicators of overall ionic concentration rather than independent effects. For spatial models, variogram analysis was used to estimate spatial correlation range. Model performance was evaluated using the following: (1) AIC comparison between spatial and non-spatial models, (2) pseudo-R^2^ for GLMMs [54], (3) residual spatial autocorrelation testing using Moran’s I on residuals, and (4) cross-validation using leave-one-site-out cross-validation (LOSO-CV). This approach is more appropriate for small sample sizes: each of the 26 sites were held out in turn as a test set while the remaining 25 sites were used for model training, resulting in 26 iterations. We acknowledge that predictive performance estimates from this approach should be interpreted cautiously, given the modest sample size.

#### Data analysis

All analyses were conducted in R 4.3.1 [55] using packages: *spatstat* for spatial point pattern analysis [56], *gstat* for geostatistics [57], *spaMM* for spatial GLMMs [58], *spdep* for spatial autocorrelation [59], and *ggplot2* for visualization [60]. Hotspot mapping and interpolation surfaces were created in QGIS 3.28 [61].

#### Ethical considerations

The study protocol was approved by the Gulu University Research Ethics Committee (GUREC-2022–323) and the Uganda National Council for Science and Technology (UNCST-HS2571ES). Community consent was obtained through local leaders before sampling. No personal identifying information was collected from individuals using water bodies.

## Results

### Snail species composition and distribution

A total of 4802 freshwater snails belonging to 13 species were collected from 26 sampling sites during both dry and rainy seasons of 2023 (supplementary file 2). The species composition and seasonal distribution are presented in Table 1. The three most abundant genera were *Biomphalaria* (47.2%, *n* = 2266), *Bulinus* (23.7%, *n* = 1138), and *Lymnaea* (10.6%, *n* = 510). *Biomphalaria choanomphala* was the most abundant species (25.8%, *n* = 1,241), followed by *Bulinus africanus* (17.7%, *n* = 852) and *Biomphalaria sudanica* (15.6%, *n* = 749).

**Table 1:** Species composition and seasonal distribution of freshwater snails in the Lango subregion, northern Uganda (*n* = 4802)

Species	Dry season (*n*)	Rainy season (*n*)	Total (*n*)	% of total
*Biomphalaria choanomphala*	794	447	1241	25.8
*Bulinus africanus*	431	421	852	17.7
*Biomphalaria sudanica*	119	630	749	15.6
*Lymnaea natalensis*	278	232	510	10.6
*Pila ovata*	125	293	418	8.7
*Lanistes carinatus*	86	272	358	7.5
*Biomphalaria pfeifferi*	187	89	276	5.7
*Bulinus ugandae*	137	105	242	5.04
*Melanoides tuberculata*	16	83	99	2.1
*Bulinus globosus*	33	1	34	0.7
*Bivalves*	13	0	13	0.3
*Bulinus forskalii*	1	6	7	0.2
*Bulinus nasutus*	3	0	3	0.1
Total	2223	2579	4802	100

Detailed site-level data, including coordinates, habitat types, and physicochemical measurements, are available in supplementary file 3 (Table S1). Seasonal analysis revealed higher overall snail abundance during the rainy season (53.7%) compared to the dry season (46.3%). However, species richness was greater in the dry season (13 species) than the rainy season (11 species). *Bulinus nasutus* and bivalves were collected exclusively during the dry season. Wilcoxon signed-rank tests confirmed significant seasonal differences in abundance for several species: *Lymnaea natalensis* was more abundant in the dry season (*Z* = − 3.064, *p* = 0.002), while *Biomphalaria choanomphala* (*Z* = − 2.076, *p* = 0.038), *B. pfeifferi* (*Z* = − 3.379, *p* = 0.001), and *Pila ovata* (*Z* = − 2.719, *p* < 0.001) were more abundant in the rainy season.

### Spatial patterns and clustering

Spatial analysis revealed significant clustering of snail populations across the study area. Global Moran’s I statistics indicated positive spatial autocorrelation for total snail density (*I* = 0.28, *p* = 0.008) and for key species: *Biomphalaria choanomphala* (*I* = 0.32, *p* = 0.004), *B. sudanica* (*I* = 0.24, *p* = 0.018), and *Bulinus africanus* (*I* = 0.19, *p* = 0.042). This suggests that snail populations are not randomly distributed but clustered in specific areas. Variogram diagnostics supporting spatial dependence are shown in Fig. 2.

Empirical semivariograms (blue points) and fitted exponential variogram models (orange curves) illustrate spatial autocorrelation in snail metrics across the georeferenced sampling sites. The x-axis displays lag distance (km), and the y-axis shows semivariance. The panels depict the following: (A) total snails collected (count), (B) *Biomphalaria choanomphala* density (snails/m^2^), (C) *Biomphalaria sudanica* density (snails/m^2^), and (D) infected snails (count) (infection status based on cercarial shedding as detailed in the Methods). Estimated exponential model parameters are as follows: (A) nugget = 1.19 × 103, partial sill = 2.58 × 104, range = 9.24 km; (B) nugget = 1.23 × 103, partial sill = 1.00 × 104, range = 13.18 km; (C) nugget = 2.33 × 10⁻11, partial sill = 2.25 × 103, range = 3.93 km; and (D) nugget = 2.3, partial sill = 0.48, range = 6.89 km. The range indicates the approximate distance over which observations remain spatially correlated.

**Fig. 3:**
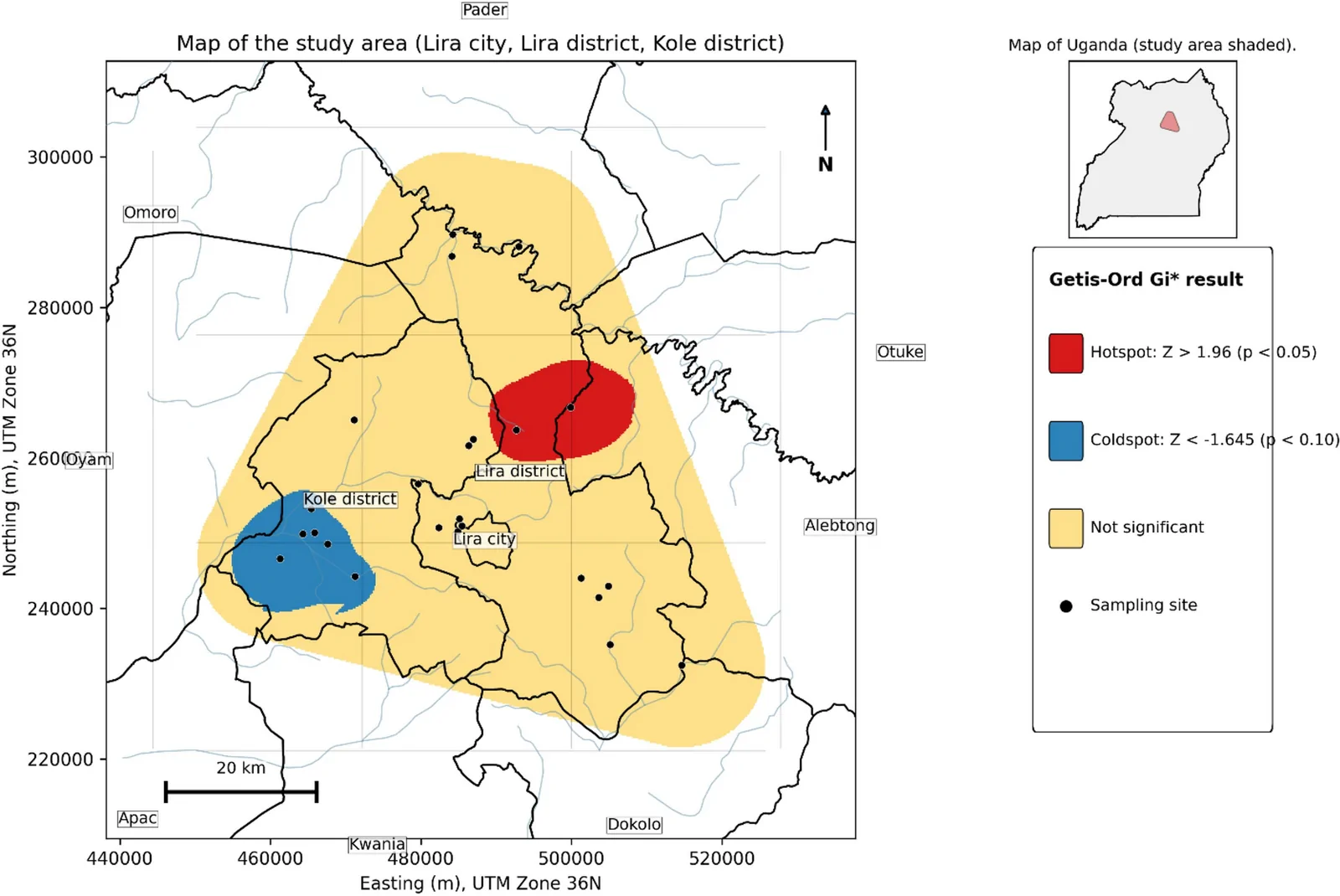
Hotspot analysis of freshwater snail density in the Lango subregion, northern Uganda

Getis-Ord Gi* hotspot analysis identified three significant clusters of high snail density (hotspots) and two coldspots (Fig. 3). The primary hotspot (Cluster A) was located in the Okile swamp and surrounding rice paddies (*Z* = 3.12, *p* = 0.002), covering approximately 4.2 km^2^. Secondary hotspots included the Aswa River confluence area (Cluster B: *Z* = 2.89, *p* = 0.004) and Apoka swamp system (Cluster C: *Z* = 2.45, *p* = 0.014). Coldspots were identified in areas with sandy substrates and high human disturbance.

Map showing Getis-Ord Gi* statistics for snail density clusters. Red areas indicate significant hotspots (*Z* > 1.96, *p* < 0.05), blue areas indicate coldspots (*Z* < − 1.645, *p* < 0.10), and yellow areas indicate non-significant zones. Sampling sites are shown as black dots. Inset shows location of study area within Uganda.

### Schistosome-like infection rates and spatial distribution

Among the 3404 *Biomphalaria* and *Bulinus* snails examined for cercarial shedding, only 5 (0.15%) were positive for patent infection (shedding schistosome-like cercariae). species-specific rates were as follows: *Biomphalaria sudanica* 0.13% (1/749), *B. choanomphala* 0.16% (2/1241), and *Bulinus africanus* 0.23% (2/852). No schistosome-like cercariae were detected in other snail species, including *Lymnaea*.

Given the small number of infected snails (*n* = 5), spatial analysis of infection is presented descriptively only. Sites where cercarial shedding was detected appeared to be clustered at one site (Abolet rice field area) (*Z* = 2.78, *p* = 0.012, though this should be interpreted descriptively given the small number of infected snails). Infected snails were collected from three distinct locations: Abolet rice field (Lira District, rainy season), Apoka swamp (Lira District, dry season), and Telela rice field (Lira City, dry season). The spatial distribution of infected snails showed possible clustering within 1.5 km radius of rice cultivation areas. However, due to the small number of the infected snails (*n* = 5), this clustering observation should be considered strictly descriptive and exploratory rather than definitive. No spatial GLMM was attempted for infection prevalence because the small number of infected snails would produce statistically unreliable estimates. Therefore, infection data are presented descriptively only.

### Physicochemical and environmental characteristics

Table 2 presents descriptive statistics for physicochemical parameters measured during the study. Water temperature ranged from 26.3 °C to 34.8 °C (mean ± SD: 29.98 ± 2.36 °C), with higher temperatures during the dry season. pH values were generally alkaline (range: 6.4–9.6, mean: 7.90 ± 0.60). Salinity was low overall (0.0–0.2 g/L, mean: 0.01 ± 0.04 g/L) but showed seasonal variation. TDS ranged widely from 42 to 906 ppm (mean: 171.46 ± 158.27 ppm), with higher values in the rainy season likely due to increased runoff. Dissolved oxygen showed considerable variation (5.0–15.6 mg/L, mean: 6.36 ± 2.87 mg/L), with higher values in the dry season reflecting increased photosynthetic activity in warmer, shallower waters.

**Table 2:** Descriptive statistics of physicochemical parameters by season

Parameter	Dry season Mean ± SD (Range)	Rainy season Mean ± SD (Range)	Overall Mean ± SD (Range)
Temperature (°C)	29.05 ± 2.12 (26.3–33.1)	30.91 ± 2.20 (27.5–34.8)	29.98 ± 2.36 (26.3–34.8)
pH	7.73 ± 0.55 (6.4–8.9)	8.08 ± 0.57 (7.1–9.6)	7.90 ± 0.60 (6.4–9.6)
Salinity (g/L)	0.02 ± 0.05 (0.0–0.2)	0.00 ± 0.01 (0.0–0.05)	0.01 ± 0.04 (0.0–0.2)
TDS (ppm)	151.54 ± 125.33 (42–580)	191.38 ± 183.56 (58–906)	171.46 ± 158.27 (42–906)
Conductivity (mS/cm)	0.24 ± 0.20 (0.01–0.75)	0.30 ± 0.35 (0.04–1.68)	0.27 ± 0.29 (0.01–1.68)
DO₂ (mg/L)	9.02 ± 2.23 (5.8–15.6)	3.69 ± 1.58 (1.6–6.9)	6.36 ± 2.87 (1.6–15.6)

Habitat characteristics varied across sites: 80.8% had muddy substrates, 53.8% had water depths of 0.5–1 m, and 40.4% had flow rates of 0.5–1 m/s. Domestic animals (primarily cattle) were present at 96.2% of sites, while water birds were observed at 67.3% of sites. Anthropogenic activities were common, with washing/bathing occurring at 65.4% of sites and rice cultivation at 30.8% of sites.

### Predictors of snail prevalence: spatial vs. non-spatial models

Table 3 presents results from logistic regression models predicting snail prevalence. For *Biomphalaria sudanica*, the spatial GLMM explained 73.1% of the variation (pseudo-R^2^ = 0.731), significantly outperforming the non-spatial GLM (ΔAIC = 15.3). Key predictors included salinity (OR = 0.21, 95% CI 0.09–0.49, p < 0.001), TDS (OR = 0.87, 95% CI 0.79–0.96, p = 0.005), conductivity (OR = 0.31, 95% CI 0.14–0.69, p = 0.004), and water depth (0.5–1 m vs. < 0.5 m: OR = 0.42, 95% CI 0.21–0.84, p = 0.014). The spatial random effect variance was 0.86, accounting for 22% of total variance.

**Table 3:** Logistic regression models for snail prevalence: comparison of spatial GLMMs and non-spatial GLMs

Species & Model	Predictor	Odds Ratio (95% CI)	*p*-value	AIC	Pseudo-R^2^
*B. sudanica* (GLM)	Salinity	0.35 (0.17–0.72)	0.004	127.4	0.521
	TDS	0.90 (0.83–0.98)	0.014		
*B. sudanica* (GLMM)	Salinity	0.21 (0.09–0.49)	< 0.001	112.1	0.731
	TDS	0.87 (0.79–0.96)	0.005		
	Conductivity	0.31 (0.14–0.69)	0.004		
	Water depth (0.5–1 m)	0.42 (0.21–0.84)	0.014		
	Spatial variance (σ^2^)	0.86			
*B. choanomphala* (GLM)	DO₂	1.12 (1.01–1.24)	0.032	98.7	0.654
	Pond habitat	3.89 (1.65–9.18)	0.002		
*B. choanomphala*(GLMM)	DO₂	1.18 (1.05–1.33)	0.006	86.0	0.822
	Pond habitat	4.56 (1.89–11.01)	< 0.001		
	Rice paddy	3.78 (1.42–10.07)	0.008		
	Washing/bathing	2.34 (1.28–4.28)	0.006		
	Car washing	0.41 (0.19–0.88)	0.022		
	Spatial variance (*σ*^2^)	1.12			

All models were fitted using *n* = 52 site-season observations. Prevalence models should be interpreted as exploratory given the modest number of positive observations (*n* = 8 for *B. sudanica*; *n* = 11 for *B. choanomphala*)

For *Biomphalaria choanomphala*, the spatial model explained 82.2% of variation (pseudo-R^2^ = 0.822, ΔAIC = 12.7). Significant predictors included dissolved oxygen (OR = 1.18, 95% CI 1.05–1.33, p = 0.006), presence in ponds (OR = 4.56, 95% CI 1.89–11.01, p < 0.001), rice paddies (OR = 3.78, 95% CI 1.42–10.07, p = 0.008), and washing/bathing activities (OR = 2.34, 95% CI 1.28–4.28, p = 0.006). Car washing showed a negative association (OR = 0.41, 95% CI 0.19–0.88, p = 0.022).

Model notes: All models were fitted using n = 52 site-season observations (26 sites × 2 seasons), with site included as random effect in GLMMs to account for the repeated measures. Prevalence was defined as site-season presence/absence of the target species. Prevalence models should be interpreted as exploratory given the modest number of positive observations (n = 8 for *B. sudanica*; n = 11 for *B. choanomphala*).

### Predictors of snail prevalence: Spatial vs. non-spatial models

Table 3 presents results from logistic regression models predicting snail prevalence. For *Biomphalaria sudanica*, the spatial GLMM explained 73.1% of the variation (pseudo-R^2^ = 0.731), significantly outperforming the non-spatial GLM (ΔAIC = 15.3). Key predictors included salinity (OR = 0.21, 95% CI 0.09–0.49, *p* < 0.001), TDS (OR = 0.87, 95% CI 0.79–0.96, *p* = 0.005), conductivity (OR = 0.31, 95% CI 0.14–0.69, *p* = 0.004), and water depth (0.5–1 m vs. < 0.5 m: OR = 0.42, 95% CI 0.21–0.84, *p* = 0.014). The spatial random effect variance was 0.86, accounting for 22% of total variance.

For *Biomphalaria choanomphala*, the spatial model explained 82.2% of variation (pseudo-R^2^ = 0.822, ΔAIC = 12.7). Significant predictors included dissolved oxygen (OR = 1.18, 95% CI 1.05–1.33, *p* = 0.006), presence in ponds (OR = 4.56, 95% CI 1.89–11.01, *p* < 0.001), rice paddies (OR = 3.78, 95% CI 1.42–10.07, *p* = 0.008), and washing/bathing activities (OR = 2.34, 95% CI 1.28–4.28, *p* = 0.006). Car washing showed a negative association (OR = 0.41, 95% CI 0.19–0.88, *p* = 0.022).

Model notes: All models were fitted using *n* = 52 site-season observations (26 sites × 2 seasons), with site included as random effect in GLMMs to account for the repeated measures. Prevalence was defined as site-season presence/absence of the target species. Prevalence models should be interpreted as exploratory given the modest number of positive observations (*n* = 8 for *B. sudanica*; *n* = 11 for *B. choanomphala*).

### Predictors of snail density: Spatial vs. non-spatial models

Table 4 presents negative binomial regression results for snail density. For *Biomphalaria sudanica*, the spatial model (ΔAIC = 14.2) identified positive associations with water temperature (IRR = 1.41, 95% CI 1.22–1.64, *p* < 0.001), pH (IRR = 2.72, 95% CI 1.76–4.20, *p* < 0.001), TDS (IRR = 1.01, 95% CI 1.00–1.01, *p* = 0.002), and washing activities (IRR = 4.62, 95% CI 2.14–9.98, *p* < 0.001). Negative associations were found with dissolved oxygen (IRR = 0.94, 95% CI 0.90–0.98, *p* = 0.003) and dry season (IRR = 0.19, 95% CI 0.09–0.40, *p* < 0.001).

**Table 4:** Negative binomial regression models for snail density: comparison of spatial GLMMs and non-spatial GLMs

Species & Model	Predictor	Incidence Rate Ratio (95% CI)	*p*-value	AIC
*B. sudanica* (GLM)	Temperature	1.32 (1.15–1.52)	< 0.001	345.2
	pH	2.45 (1.62–3.70)	< 0.001	
	Washing activity	3.85 (1.85–8.01)	< 0.001	
*B. sudanica* (GLMM)	Temperature	1.41 (1.22–1.64)	< 0.001	331.0
	pH	2.72 (1.76–4.20)	< 0.001	
	TDS	1.01 (1.00–1.01)	0.002	
	DO₂	0.94 (0.90–0.98)	0.003	
	Washing activity	4.62 (2.14–9.98)	< 0.001	
	Dry season	0.19 (0.09–0.40)	< 0.001	
	Spatial variance (*σ*^2^)	0.74		
*B. choanomphala* (GLM)	DO₂	1.05 (1.01–1.09)	0.012	312.8
	Stream habitat	4.12 (2.01–8.45)	< 0.001	
*B. choanomphala* (GLMM)	DO₂	1.07 (1.03–1.11)	< 0.001	299.1
	Stream habitat	5.07 (2.44–10.53)	< 0.001	
	Temperature	0.50 (0.38–0.66)	< 0.001	
	TDS	0.96 (0.94–0.98)	< 0.001	
	Conductivity	0.28 (0.15–0.52)	< 0.001	
	Spatial variance (*σ*^2^)	0.89		

All models fitted using *n* = 52 site-season observations

For *Biomphalaria choanomphala*, density increased with dissolved oxygen (IRR = 1.07, 95% CI 1.03–1.11, *p* < 0.001) and presence in streams (IRR = 5.07, 95% CI 2.44–10.53, *p* < 0.001), but decreased with temperature (IRR = 0.50, 95% CI 0.38–0.66, *p* < 0.001), TDS (IRR = 0.96, 95% CI 0.94–0.98, *p* < 0.001), and conductivity (IRR = 0.28, 95% CI 0.15–0.52, *p* < 0.001).

Model notes: All models were fitted using *n* = 52 site-season observations (26 sites × 2 seasons), with site included as random effect in GLMMs to account for the repeated measures. Density was defined as snails/m^2^, calculated as: number of snails collected per site-season ÷ 5 m^2^ sampled area. Values are best interpreted as standardized relative abundance or catch-per-unit-effort (CPUE).

### Spatial interpolation of key parameters

**Fig. 4:**
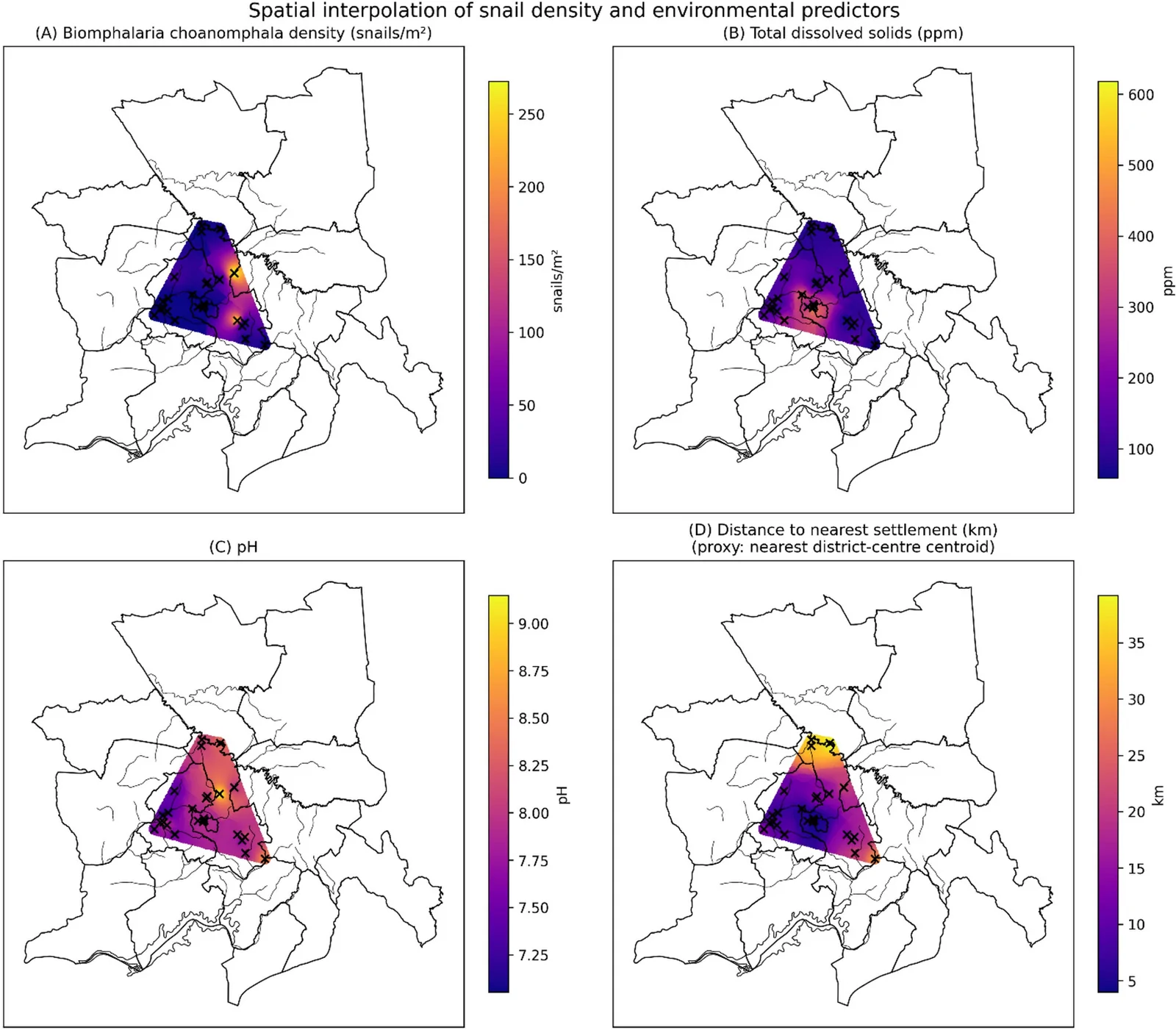
Spatial interpolation of snail density and environmental predictors Four-panel figure showing inverse distance weighting interpolation surfaces for the following: ***A**** Biomphalaria choanomphala density (snails/m*^*2*^*), ****B**** Total dissolved solids (ppm), ****C**** pH, and (****D****) Distance to nearest settlement (km).* Warmer colors indicate higher values. Cross symbols show sampling locations

Figure 4 presents IDW interpolation surfaces for *Biomphalaria choanomphala* density and key environmental predictors. High-density areas corresponded with regions of moderate TDS (150–250 ppm), neutral to slightly alkaline pH (7.5–8.0), and proximity to human settlements. The interpolation revealed a gradient of decreasing snail density from central wetland areas toward peripheral regions with sandy substrates and lower vegetation cover.

### Model validation and performance

Spatial models consistently outperformed non-spatial counterparts across all response variables (mean ΔAIC = 13.4, range: 8.3–18.1). Residual spatial autocorrelation was significantly reduced in spatial models (Moran’s I on residuals: − 0.03 to 0.11, *p* > 0.05) compared to non-spatial models (Moran’s *I*: 0.24–0.38, *p* < 0.05). Leave-one-site-out cross-validation (LOSO-CV) showed improved indicative predictive performance for spatial models (mean absolute error reduction: 18–32% across models). These estimates should be interpreted cautiously, given the modest sample size (*n* = 26 sites).

## Discussion

This study represents one of the first comprehensive integrations of geostatistical analysis with ecological modeling of freshwater snail intermediate hosts in northern Uganda. Our findings demonstrate significant spatial structuring of snail populations, with clear hotspots of high density and sites where schistosome-like cercarial shedding was detected that traditional non-spatial approaches would miss. The spatial GLMMs consistently outperformed their non-spatial counterparts, explaining 18–24% more variance by incorporating spatial random effects.

The overall patent infection rate (0.15%) is lower than rates reported from the Lake Victoria basin (0.5–2.8%) [62, 63], but it aligns with findings from other inland regions of Uganda [64, 65]. However, this rate is based on cercarial shedding without molecular confirmation; true infection rates may be higher if the prepatent infections were present but not detected by shedding tests. Additionally, while cercarial morphology was consistent with mammalian schistosomes, we cannot definitely distinguish between human-infective and non-human schistosome species without molecular methods. The low observed infection rate raises questions about whether it reflects naturally low transmission season, successful mass drug administration (MDA) programs, or the inherent limitations of cercarial shedding for detecting prepatent infections. Lira and Kole districts have received regular MDA through the Uganda Ministry of Health’s schistosomiasis control program, which may have reduced human prevalence and consequently snail infection rates. However, cross-sectional studies cannot distinguish between these possibilities. Longitudinal sampling coupled with molecular detection methods would be needed to determine whether this low rate represents sustained transmission reduction or seasonal fluctuation [66, 67]. The apparent clustering of sites where cercarial shedding was detected in rice paddies near settlements emphasizes the importance of agricultural water management in creating potential transmission sites, consistent with studies from Tanzania and Kenya [68, 69]. However, with only five infected snails, this observation is strictly descriptive and exploratory.

The identified density hotspots in Okile swamp, Aswa river confluence, and Apoka swamp exhibit common features: moderate TDS (150–250 ppm), neutral to slightly alkaline pH (7.5–8.0), muddy substrates, and proximity to human settlements with frequent water contact activities. These conditions match the known ecological preferences of *Biomphalaria* and *Bulinus* species [70, 71]. The negative correlation between car washing and snail prevalence/density suggests that either chemical pollutants or physical disturbance from this activity may harm snail habitats, or that car washing sites coincide with unfavorable physical conditions such as higher flow velocities. We cannot rule out that water flow velocity, rather than chemical pollutants, explains this association. Car washing activities typically occur in areas with easy water access, which often coincide with shallower, slower-flowing sections that may be suitable for certain snail species regardless of washing activities. While we measured flow velocity categorically (0.5–1 m/s), finer-scale velocity measurements might reveal confounding effects. Further studies should measure velocity continuously and include water quality parameters specific to car washing runoff (e.g., detergents, heavy metals) [72, 73].

Seasonal variations in snail abundance followed expected patterns, with higher densities during the rainy season due to expanded habitats and increased nutrient inputs [74, 75]. We acknowledge an alternative perspective: in lotic (flowing) systems, heavy rains can decrease snail density through flushing effects that physically remove snails from substrates. However, the majority of our sampling sites (80.8%) were lentic (still water) habitats, ponds, swamps, and rice paddies, where flushing is minimal. The observed rainy season increase in our study area likely reflects habitat expansion and nutrient inputs in these lentic systems rather than contradicting the flushing effect observed in riverine systems. However, the greater species richness during the dry season may reflect concentration effects as water bodies contract, making snails easier to sample. This finding has implications for control timing: dry-season interventions may be strategically advantageous because (1) snail populations are concentrated in the remaining water bodies, making them easier to locate and target; (2) chemical mollusciciding may be more effective when water volumes are lower; (3) habitat modification (e.g., draining or drying rice paddies) can be implemented during the dry season to disrupt snail life cycles. However, we caution that during dry-season aestivation (when snails burrow into the mud to survive desiccation), standard mollusciciding of surface water may miss aestivating snails. Therefore, dry-season interventions should consider environmental modification (e.g., plowing or desiccating rice paddies) rather than relying solely on chemical control.

The spatial autocorrelation detected for key species (Moran’s *I* = 0.19–0.32) indicates that snail populations are not randomly distributed but exhibit patchiness at scales of 1–3 km. This clustering likely results from both environmental heterogeneity (habitat suitability gradients) and limited dispersal capabilities of snails [76, 77]. Similar spatial patterns have been reported for snail populations in Kenya and Tanzania [78, 79].

Anthropogenic activities exhibited complex spatial relationships with snail distributions. Washing and bathing activities generally increased snail density, possibly due to nutrient enrichment from soap and organic matter [80, 81]. Conversely, car washing and swimming/playing were linked to adverse effects, likely owing to habitat disturbance and chemical inputs [82]. These findings highlight the importance of context-specific interventions that consider local water-use practices.

The superior performance of spatial models (ΔAIC = 8.3–18.1) highlights the importance of considering spatial autocorrelation in ecological studies of snail distributions. Ignoring spatial dependencies can result in biased parameter estimates, increased Type I errors, and poor predictions for targeted control [83, 84]. Our spatial GLMMs effectively captured both fixed effects of environmental predictors and random spatial effects, offering more reliable inference.

### Study limitations

Several limitations should be acknowledged when interpreting our findings. First, the cross-sectional design offers only a snapshot of snail distributions and infection rates. Long-term sampling over multiple years would better capture temporal dynamics and seasonal changes. Second, although we included a comprehensive range of environmental predictors, unmeasured factors such as predation pressure, competition, and microhabitat characteristics could influence snail distributions. Third, the moderate spatial resolution (26 sites across approximately 2000 km^2^) may not capture fine-scale heterogeneity, especially in complex wetland systems. However, our stratified random sampling was explicitly designed to capture major habitat types across the study area, and the hotspot analysis successfully identified significant risk clusters despite this potential limitation. Fourth, cercarial shedding has limited sensitivity for detecting prepatent infections, which might lead to an underestimation of true infection rates. Additionally, infection detection relied on cercarial shedding without molecular confirmation, which may underestimate true infection rates and limit species-specific identification of schistosomes. Molecular methods would enable more accurate detection of infections [85, 86]. Fifth, with only five infected snails detected, our infection-related findings (including spatial clustering) are strictly descriptive and exploratory; no reliable statistical modeling of infection predictors was possible. Sixth, the logistic regression models for prevalence should be interpreted with caution given the modest number of positive observations for the target species, which limited our ability to include a larger set of predictors. Finally, our study area reflects the specific ecological conditions of the Lango subregion; therefore, findings might not be directly generalizable to regions with different hydrological or land use features.

### Implications for control

The spatial density hotspots identified in this study could serve as priority areas for further surveillance and pilot studies to evaluate potential snail control measures. These suggestions require validation through longitudinal studies before implementation at scale. Combining approaches such as environmental modification (habitat disturbance in high-density zones), chemical control (targeted mollusciciding), biological control (duck introduction in rice paddies), and community-based sanitation improvements could be considered in these high-risk zones pending further validation [87, 88]. The spatial risk maps produced could potentially assist district health authorities in planning targeted mass drug administration and health education campaigns, but should be validated with human prevalence data first [89, 90].

It is crucial to distinguish between snail density hotspots (representing potential transmission risk areas where conditions favor snail populations) and sites where infected snails were detected (which may indicate active transmission, though this requires confirmation with only five infected snails). Our study primarily identifies the former; infection data are presented descriptively and should be interpreted with caution.

Future research should focus on the following: (1) longitudinal monitoring of identified density hotspots to understand transmission dynamics, (2) experimental manipulation of key environmental factors to test causality, (3) integration of remote sensing data for larger-scale habitat mapping, and (4) community participatory approaches to develop context-appropriate interventions [91–93].

## Conclusions

This study highlights the added value of geostatistical approaches in understanding the spatial ecology of schistosomiasis intermediate host snails in northern Uganda. The use of spatial clustering analysis, hotspot mapping, and spatial regression modeling identified distinct, high-risk snail density clusters mainly located in rice paddies and swamps near human settlements, which would be overlooked by traditional non-spatial methods. The demonstrated effectiveness of spatial models, combined with key environmental and anthropogenic predictors, provides preliminary evidence that could guide further surveillance and consideration of targeted control measures. It is crucial to distinguish between snail density hotspots (representing potential transmission risk areas where conditions favor snail populations) and sites where infected snails were detected (which may indicate active transmission, though this requires confirmation with only five infected snails). Our study primarily identifies the former; infection data are presented descriptively and should be interpreted with caution. Our findings support the consideration of spatially explicit interventions, such as targeted snail control in high-density areas pending validation studies, community-led habitat modification, and the integration of risk maps into health planning, which could improve the efficiency of schistosomiasis control efforts in Uganda and similar endemic areas. Findings should be validated through longitudinal studies and pilot interventions before programmatic implementation.

## Supplementary Information

Additional file 1.

Additional file 2.

Additional file 3.

Additional file 4.

Additional file 5.

## Data Availability

All data generated or analyzed during this study are included in this published article and its supplementary information files. A complete analyzable dataset containing all variables used in the final and candidate models, including the corrected dissolved oxygen values, is provided as Supplementary file 4 (Table S2).

## Abbreviations

AIC: Akaike Information Criterion
CI: Confidence Interval
CPUE: Catch-Per-Unit-Effort
DO₂: Dissolved Oxygen
GLM: Generalized Linear Model
GLMM: Generalized Linear Mixed Model
GPS: Global Positioning System
IDW: Inverse Distance Weighting
IRR: Incidence Rate Ratio
LOSO-CV: Leave-One-Site-Out Cross-Validation
OR: Odds Ratio
SD: Standard Deviation
TDS: Total Dissolved Solids
VIF: Variance Inflation Factor

## References

[1] Brown DS. Freshwater snails of Africa and their medical importance. 2nd ed. London: Taylor & Francis; 1994.

[2] Colley DG, Bustinduy AL, Secor WE, King CH. Human schistosomiasis. Lancet. 2014;383:2253–64. 10.1016/S0140-6736(13)61949-2 PMC4672382 24698483

[3] World Health Organization. Schistosomiasis: key facts. Geneva: World Health Organization; 2022.

[4] Adriko M, Tinkitina B, Tukahebwa EM, Standley CJ, Stothard JR, Kabatereine NB. The epidemiology of schistosomiasis in Lango region Uganda 60 years after Schwetz 1951. Acta Trop. 2018;185:412–8. 10.1016/j.actatropica.2018.06.009 29935144

[5] Ministry of Health Uganda. Uganda national master plan for neglected tropical diseases 2017–2022. Kampala: Ministry of Health Uganda; 2017.

[6] Nalugwa A, Nuwaha F, Tukahebwa EM, Olsen A. Single versus double dose praziquantel comparison on efficacy and *schistosoma mansoni* re-infection in preschool-age children in Uganda: a randomized controlled trial. BMC Infect Dis. 2015;15:357. 10.1371/journal.pntd.0003796 PMC4444284 26011733

[7] Ezeamama AE, Bustinduy AL, Nkwata AK, Martinez L, Pabalan N, Boivin MJ, et al. Cognitive deficits and educational loss in children with schistosome infection-a systematic review and meta-analysis. PLoS Negl Trop Dis. 2018;12:e0005524. 10.1371/journal.pntd.0005524 PMC5766129 29329293

[8] Rollinson D, Knopp S, Levitz S, Stothard JR, Tchuem Tchuenté LA, Garba A, et al. Time to set the agenda for schistosomiasis elimination. Acta Trop. 2013;128:423–40. 10.1016/j.actatropica.2012.04.013 22580511

[9] Mas-Coma S, Valero MA, Bargues MD. Fascioliasis. Adv Exp Med Biol. 2019;1154:71–103. 10.1007/978-3-030-18616-6_4 31297760

[10] Appleton CC. Review of literature on abiotic factors influencing the distribution and life cycles of bilharziasis intermediate host snails. Malacol Rev. 1978;11:1–25.

[11] Kloos H, Passos LK, LoVerde P, Oliveira RC, Gazzinelli A. The role of population movement in the epidemiology and control of schistosomiasis in Brazil: a preliminary typology of population movement. Mem Inst Oswaldo Cruz. 2010;105:578–86. 10.1590/s0074-02762010000400038 20721511

[12] Standley CJ, Adriko M, Arinaitwe M, Atuhaire A, Kazibwe F, Fenwick A, et al. Epidemiology and control of intestinal schistosomiasis on the Sesse Islands, Uganda: integrating malacology and parasitology to tailor local treatment recommendations. Parasit Vectors. 2010;3:64. 10.1186/1756-3305-3-64 PMC2920240 20663223

[13] Kazibwe F, Makanga B, Rubaire-Akiiki C, Ouma J, Kariuki C, Kabatereine NB, et al. Ecology of *Biomphalaria* (Gastropoda: Planorbidae) in Lake Albert, Western Uganda: snail distributions, infection with schistosomes and temporal associations with environmental dynamics. Hydrobiologia. 2006;568:433–44.

[14] Andrus PS, Stothard JR, Wade CM. Seasonal patterns of Schistosoma mansoni infection within Biomphalaria snails at the Ugandan shorelines of Lake Albert and Lake Victoria. *PLoS Neglected Tropical Diseases*. 2023 Aug 14;17(8): e0011506. 10.1371/journal.pntd.0011506. PMC10424865 37578945

[15] Ejotre I, Makanga B, Nachuha S, Mpezamihigo M. Environmental parameters and *Biomphalaria* snail distribution along River Kochi, West Nile Region. Uganda Adv Res. 2015;3:244–50.

[16] Byagamy JP, Malinga GM, Angwech H, Opiro R, Echodu R, Odongo-Aginya E. Prevalence, infection intensity and associated risk factors for intestinal schistosomiasis among primary school children in Lira District. Northern Uganda Sci Rep. 2020;10:15429.

[17] Uganda Bureau of Statistics. National population and housing census 2014 area specific profiles - Lira district. Kampala: Uganda Bureau of Statistics; 2017.

[18] National Environment Management Authority. State of the environment report for Uganda 2018. Kampala: National Environment Management Authority; 2019.

[19] Tumusiime J, Kagoro-Rugunda G, Mbabazi J, Hakizimana J. Impact of rice irrigation on malaria transmission in Lango sub-region. Uganda Malar J. 2020;19:280.

[20] Steinmann P, Keiser J, Bos R, Tanner M, Utzinger J. Schistosomiasis and water resources development: systematic review, meta-analysis, and estimates of people at risk. Lancet Infect Dis. 2006;6:411–25. 10.1016/S1473-3099(06)70521-7 16790382

[21] Ofulla AV, Adoka SO, Anyona DN, Abuom PO, Karanja D, Vulule JM, et al. Spatial distribution and habitat characterization of schistosomiasis host snails in lake and land habitats of western Kenya. Lakes Reserv Sci Policy Manag Sustain Use. 2013;18:197–215.

[22] Oso OG, Odaibo AB. Land use/land cover change, physicochemical parameters and freshwater snails in Yewa North, Southwestern Nigeria. PLoS ONE. 2021;16:e0245678. 10.1371/journal.pone.0246566 PMC7870010 33556093

[23] Min F, Wang J, Liu X, Yuan Y, Guo Y, Zhu K, et al. Environmental factors affecting freshwater snail intermediate hosts in Shenzhen and adjacent region, South China. Trop Med Infect Dis. 2022;7:401. 10.3390/tropicalmed7120426 PMC9782201 36548681

[24] Brooker S, Hay SI, Bundy DA. Tools from ecology: useful for evaluating infection risk models? Trends Parasitol. 2002;18:70–4. 10.1016/s1471-4922(01)02223-1 PMC3166848 11832297

[25] Sturrock HJ, Gething PW, Ashton RA, Kolaczinski JH, Kabatereine NB, Brooker S. Planning schistosomiasis control: investigation of alternative sampling strategies for *Schistosoma mansoni* to target mass drug administration of praziquantel in East Africa. Int Health. 2011;3:165–75. 10.1016/j.inhe.2011.06.002 24038366

[26] Dormann CF, Elith J, Bacher S, Buchmann C, Carl G, Carré G, et al. Collinearity: a review of methods to deal with it and a simulation study evaluating their performance. Ecography. 2013;36:27–46.

[27] Beale CM, Lennon JJ, Elston DA, Brewer MJ, Yearsley JM. Regression analysis of spatial data. Ecol Lett. 2007;10:997–1005. 10.1111/j.1461-0248.2009.01422.x 20102373

[28] Diggle PJ, Ribeiro PJ. Model-based geostatistics. New York: Springer; 2007.

[29] Haining R. Spatial data analysis: theory and practice. Cambridge: Cambridge University Press; 2003.

[30] Lawson AB. Statistical methods in spatial epidemiology. 2nd ed. Chichester: Wiley; 2006.

[31] Manyangadze T, Chimbari MJ, Gebreslasie M, Ceccato P, Mukaratirwa S. Modelling the spatial and seasonal distribution of suitable habitats of schistosomiasis intermediate host snails using Maxent in Ndumo area, KwaZulu-Natal Province. South Africa Parasit Vectors. 2016;9:572. 10.1186/s13071-016-1834-5 PMC5096326 27814746

[32] Gouvras AN, Allan F, Kinung’hi S, Rabone M, Emery A, Angelo T, et al. Longitudinal survey on the distribution of *Biomphalaria sudanica* and *B. choanomphala* in Mwanza region, on the shores of Lake Victoria, Tanzania: implications for schistosomiasis transmission and control. Parasit Vectors. 2017;10:316. 10.1186/s13071-017-2252-z PMC5490224 28659165

[33] Babbitt CR, Laidemitt MR, Mutuku MW, Oraro PO, Brant SV, Mkoji GM, Loker ES. Bulinus snails in the Lake Victoria Basin in Kenya: Systematics and their role as hosts for schistosomes. *PLOS Neglected Tropical Diseases*. 2023 Feb 10;17(2): e0010752 10.1371/journal.pntd.0010752. PMC9949660 36763676

[34] Nwoko OE, Manyangadze T, Chimbari MJ. Spatial distribution, abundance, and infection rates of human schistosome-transmitting snails and related physicochemical parameters in KwaZulu-Natal (KZN) province, South Africa. Heliyon. 2023;9:e12463. 10.1016/j.heliyon.2022.e12463 PMC9922783 36793949

[35] Knopp S, Person B, Ame SM, Ali SM, Hattendorf J, Juma S, et al. Evaluation of integrated interventions layered on mass drug administration for urogenital schistosomiasis elimination: a cluster-randomised trial. Lancet Glob Health. 2019;7:e1118–29. 10.1016/S2214-109X(19)30189-5 PMC6624424 31255591

[36] Toor J, Alsallaq R, Truscott JE, Turner HC, Werkman M, Gurarie D, et al. Are we on our way to achieving the 2020 goals for schistosomiasis morbidity control using current World Health Organization guidelines? Clin Infect Dis. 2020;66:S245–52. 10.1093/cid/ciy001 PMC5982704 29860290

[37] World Health Organization. Ending the neglect to attain the sustainable development goals: a road map for neglected tropical diseases 2021-2030. Geneva: World Health Organization; 2020.

[38] Basalirwa CPK. Delineation of Uganda into climatological rainfall zones using the method of principal component analysis. Int J Climatol. 1995;15:1161–77.

[39] National Meteorological Authority. Climate of Uganda. Kampala: National Meteorological Authority; 2021.

[40] Ministry of Water and Environment. Wetlands atlas of Uganda. Kampala: Ministry of Water and Environment; 2016.

[41] Uganda Bureau of Statistics. Statistical abstract 2022. Kampala: Uganda Bureau of Statistics; 2022.

[42] Appleton C, Miranda N. Locating bilharzia transmission sites in South Africa: guidelines for public health personnel. S Afr J Infect Dis. 2015;30:95–102.

[43] Mandahl-Barth G. Identification key to the freshwater snails of the Ethiopian region. Charlottenlund: Danish Bilharziasis Laboratory; 1988.

[44] Frandsen F, Christensen NO. An introductory guide to the identification of cercariae from African freshwater snails with special reference to cercariae of trematode species of medical and veterinary importance. Acta Trop. 1984;41:181–202. 6206702

[45] Yigezu G, Mandefro B, Mengesha Y, Yewhalaw D, Beyene A, Ahmednur M, et al. Habitat suitability modelling for predicting potential habitats of freshwater snail intermediate hosts in Omo-Gibe river basin. Southwest Ethiopia Ecol Inform. 2018;45:70–80.

[46] Kalinda C, Chimbari M, Mukaratirwa S. Implications of changing temperatures on the growth, fecundity and survival of intermediate host snails of schistosomiasis: a systematic review. Int J Environ Res Public Health. 2017;14:80. 10.3390/ijerph14010080 PMC5295331 28098789

[47] Moran PAP. Notes on continuous stochastic phenomena. Biometrika. 1950;37:17–23. 15420245

[48] Woolhouse MEJ, Chandiwana SK. Spatial and temporal heterogeneity in the population dynamics of *Bulinus globosus* and *Biomphalaria pfeifferi* and in the epidemiology of their infection with schistosomes. Parasitology. 1989;98:21–34. 10.1017/s0031182000059655 2717216

[49] Getis A, Ord JK. The analysis of spatial association by use of distance statistics. Geogr Anal. 1992;24:189–206.

[50] Shepard D. A two-dimensional interpolation function for irregularly-spaced data. In: Proceedings of the 1968 23rd ACM national conference. 1968. p. 517–24.

[51] Breslow NE, Clayton DG. Approximate inference in generalized linear mixed models. J Am Stat Assoc. 1993;88:9–25.

[52] Akaike H. A new look at the statistical model identification. IEEE Trans Autom Control. 1974;19:716–23.

[53] O’Brien RM. A caution regarding rules of thumb for variance inflation factors. Qual Quant. 2007;41:673–90.

[54] Nakagawa S, Schielzeth H. A general and simple method for obtaining R2 from generalized linear mixed-effects models. Methods Ecol Evol. 2013;4:133–42.

[55] R Core Team. R: a language and environment for statistical computing. Vienna: R Foundation for Statistical Computing; 2023.

[56] Baddeley A, Rubak E, Turner R. Spatial point patterns: methodology and applications with R. Boca Raton: Chapman and Hall/CRC; 2015.

[57] Pebesma EJ. Multivariable geostatistics in S: the gstat package. Comput Geosci. 2004;30:683–91.

[58] Rousset F, Ferdy JB. Testing environmental and genetic effects in the presence of spatial autocorrelation. Ecography. 2014;37:781–90.

[59] Bivand RS, Pebesma E, Gómez-Rubio V. Applied spatial data analysis with R. 2nd ed. New York: Springer; 2013.

[60] Wickham H. ggplot2: elegant graphics for data analysis. New York: Springer; 2016.

[61] QGIS Development Team. QGIS geographic information system. Open Source Geospatial Foundation; 2023.

[62] Trienekens SCM, Faust CL, Besigye F, Pickering L, Tukahebwa EM, Seeley J, et al. Variation in water contact behaviour and risk of *Schistosoma mansoni* (re)infection among Ugandan school-aged children in an area with persistent high endemicity. Parasit Vectors. 2022;15:141. 10.1186/s13071-021-05121-6 PMC8734346 34991702

[63] Moses A, Adriko M, Kibwika B, Tukahebwa EM, Faust CL, Lamberton PHL. Residence time, water contact, and age-driven *Schistosoma mansoni* infection in hotspot communities in Uganda. Am J Trop Med Hyg. 2021;105:1772–81. 10.4269/ajtmh.21-0391 PMC8641335 34662868

[64] Katungi A, Redeker S, Kiiza P, Ocaka J, Van Goor A, Polderman AM, et al. Reassessment of helminth infections in Gulu municipality northern Uganda after the twenty years of insurgency: using three diagnostic methods to compare their sensitivity. East Afr Med J. 2013;90:95–103. 26866108

[65] Imran E. Prevalence and intensity of schistosomiasis in adjacent human communities along the River Kochi, West Nile Region of Uganda. Int J Trop Dis Health. 2014;4:729–39.

[66] Lu L, Zhang SM, Mutuku MW, Mkoji GM, Loker ES. Relative compatibility of Schistosoma mansoni with *Biomphalaria sudanica* and *B. pfeifferi* from Kenya as assessed by PCR amplification of the *S. mansoni* ND5 gene in conjunction with traditional methods. Parasit Vectors. 2016;9:1668. 10.1186/s13071-016-1457-x PMC4802880 27000855

[67] Allan F, Dunn AM, Emery AM, Stothard JR, Johnston DA, Kane RA, et al. Use of sentinel snails for the detection of *Schistosoma haematobium* transmission on Zanzibar and observations on transmission patterns. Acta Trop. 2013;128:234–40. 10.1016/j.actatropica.2013.01.003 23318933

[68] Fuss A, Mazigo HD, Mueller A. Malacological survey to identify transmission sites for intestinal schistosomiasis on Ijinga Island, Mwanza, north-western Tanzania. Acta Trop. 2020;203:105309. 10.1016/j.actatropica.2019.105289 31837312

[69] Dida GO, Gelder FB, Anyona DN, Matano AS, Abuom PO, Adoka SO, et al. Distribution and abundance of schistosomiasis and fascioliasis host snails along the Mara River in Kenya and Tanzania. Infect Ecol Epidemiol. 2014;4:24281. 10.3402/iee.v4.24281 PMC4216393 25405008

[70] Utzinger J, Tanner M. Microhabitat preferences of *Biomphalaria pfeifferi* and *Lymnaea natalensis* in a natural and a man-made habitat in southeastern Tanzania. Mem Inst Oswaldo Cruz. 2000;95:287–94. 10.1590/s0074-02762000000300002 10800185

[71] Oloyede OO, Otarigho B, Morenikeji O. Diversity, distribution and abundance of freshwater snails in Eleyele dam, Ibadan, south-west Nigeria. Zool Ecol. 2017;27:35–43.

[72] Usman AI, Adamu T, Abdulhamid A. Studies on distribution and abundance of freshwater snail intermediate hosts of schistosomiasis along Kwanar Areh Dam in Rimi L.G.A. of Katsina State. J Parasitol Vector Biol. 2019;11:26–35.

[73] Nwachukwu MO, Azorji JN, Ukaoma AA, Onyebuagu PC, Izundu MI. Evaluating the ecology and distribution of snail hosts of *Schistosoma* at the water bodies in Ihitte-Uboma area of Imo State. Adv Res. 2020;21:52–64.

[74] McCreesh N, Booth M. Challenges in predicting the effects of climate change on *Schistosoma mansoni* and *Schistosoma haematobium* transmission potential. Trends Parasitol. 2013;29:548–55. 10.1016/j.pt.2013.08.007 24064438

[75] Kalinda C, Chimbari M, Mukaratirwa S. Effect of temperature on the Bulinus globosus - *Schistosoma haematobium* system. Infect Dis Poverty. 2017;6:57. 10.1186/s40249-017-0260-z PMC5410706 28457230

[76] Stensgaard AS, Utzinger J, Vounatsou P, Hürlimann E, Schur N, Saarnak CF, et al. Large-scale determinants of intestinal schistosomiasis and intermediate host snail distribution across Africa: does climate matter? Acta Trop. 2013;128:378–90. 10.1016/j.actatropica.2011.11.010 22142789

[77] Pedersen UB, Stendel M, Midzi N, Mduluza T, Soko W, Stensgaard AS, et al. Modelling climate change impact on the spatial distribution of fresh water snails hosting trematodes in Zimbabwe. Parasit Vectors. 2014;7:536. 10.1186/s13071-014-0536-0 PMC4299310 25498001

[78] Hamburger J, Hoffman O, Kariuki HC, Muchiri EM, Ouma JH, Koech DK, et al. Large-scale, polymerase chain reaction-based surveillance of *Schistosoma haematobium* DNA in snails from transmission sites in coastal Kenya: a new tool for studying the dynamics of snail infection. Am J Trop Med Hyg. 2004;71:765–73. 15642969

[79] Pennance T, Person B, Muhsin MA, Khamis AN, Muhsin J, Khamis IS, et al. Urogenital schistosomiasis transmission on Unguja Island, Zanzibar: characterisation of persistent hot-spots. Parasit Vectors. 2016;9:646. 10.1186/s13071-016-1847-0 PMC5162088 27986092

[80] Grimes JET, Croll D, Harrison WE, Utzinger J, Freeman MC, Templeton MR. The roles of water, sanitation and hygiene in reducing schistosomiasis: a review. Parasit Vectors. 2015;8:156. 10.1186/s13071-015-0766-9 PMC4377019 25884172

[81] Freeman MC, Ogden S, Jacobson J, Abbott D, Addiss DG, Amynie AG, et al. Integration of water, sanitation, and hygiene for the prevention and control of neglected tropical diseases: a rationale for inter-sectoral collaboration. PLoS Negl Trop Dis. 2013;7:e2439. 10.1371/journal.pntd.0002439 PMC3784463 24086781

[82] Archer J, O’Halloran L, Al-Shehri H, Summers S, Bhattacharyya T, Kabatereine NB, et al. A scoping review of the role of environmental factors on the transmission of schistosomiasis in sub-Saharan Africa. PLoS Negl Trop Dis. 2023;17:e0011182.

[83] Dormann CF. Effects of incorporating spatial autocorrelation into the analysis of species distribution data. Glob Ecol Biogeogr. 2007;16:129–38.

[84] Miller JA. Species distribution modeling. Geogr Compass. 2010;4:490–509.

[85] Hamburger J, Xu Y, Ramzy RM, Jourdane J, Ruppel A. Development and laboratory evaluation of a polymerase chain reaction for monitoring *Schistosoma mansoni* infestation of water. Am J Trop Med Hyg. 1998;59:468–73. 10.4269/ajtmh.1998.59.468 9749646

[86] Gandasegui J, Fernández-Soto P, Hernández-Goenaga J, López-Abán J, Vicente B, Muro A. Biompha-LAMP: a new rapid loop-mediated isothermal amplification assay for detecting *Schistosoma mansoni* in *Biomphalaria glabrata* snail host. PLoS Negl Trop Dis. 2016;10:e0005225. 10.1371/journal.pntd.0005225 PMC5179122 27941967

[87] Sokolow SH, Wood CL, Jones IJ, Swartz SJ, Lopez M, Hsieh MH, et al. Global assessment of schistosomiasis control over the past century shows targeting the snail intermediate host works best. PLoS Negl Trop Dis. 2016;10:e0004794. 10.1371/journal.pntd.0004794 PMC4956325 27441556

[88] King CH, Sutherland LJ, Bertsch D. Systematic review and meta-analysis of the impact of chemical-based mollusciciding for control of *Schistosoma mansoni* and *S. haematobium* transmission. PLoS Negl Trop Dis. 2015;9:e0004290. 10.1371/journal.pntd.0004290 PMC4692485 26709922

[89] Campbell SJ, Savage GB, Gray DJ, Atkinson JA, Soares Magalhães RJ, Nery SV, et al. Water, sanitation, and hygiene (WASH): a critical component for sustainable soil-transmitted helminth and schistosomiasis control. PLoS Negl Trop Dis. 2014;8:e2651. 10.1371/journal.pntd.0002651 PMC3983087 24722335

[90] Campbell CH, Biritwum NK, Woods G, Velleman Y, Fleming F, Stothard JR. Tailoring water, sanitation, and hygiene (WASH) targets for soil-transmitted helminthiasis and schistosomiasis control. Trends Parasitol. 2018;34:53–63. 10.1016/j.pt.2017.09.004 29055522

[91] Knopp S, Ame SM, Hattendorf J, Ali SM, Khamis IS, Bakar F, et al. Urogenital schistosomiasis elimination in Zanzibar: accuracy of urine filtration and haematuria reagent strips for diagnosing light intensity *Schistosoma haematobium* infections. Parasit Vectors. 2018;11:552. 10.1186/s13071-018-3136-6 PMC6199745 30352631

[92] Garchitorena A, Sokolow SH, Roche B, Ngonghala CN, Jocque M, Lund A, et al. Disease ecology, health and the environment: a framework to account for ecological and socio-economic drivers in the control of neglected tropical diseases. Philos Trans R Soc Lond B Biol Sci. 2017;372:20160128. 10.1098/rstb.2016.0128 PMC5413876 28438917

[93] Bärenbold O, Garba A, Colley DG, Fleming FM, Haggag AA, Ramzy RMR, et al. Translating preventive chemotherapy prevalence thresholds for *Schistosoma mansoni* from the Kato-Katz technique into the point-of-care circulating cathodic antigen diagnostic test. PLoS Negl Trop Dis. 2018;12:e0006941. 10.1371/journal.pntd.0006941 PMC6310297 30550594

