# Supplementary material for: Spatio-ecological determinants of Biomphalaria and Bulinus snail intermediate hosts and schistosome-like infections in the Lango subregion, Northern Uganda: a geostatistical approach to guide targeted disease control
Source: Parasit Vectors. 2026 Aug 18;19:492. doi: 10.1186/s13071-026-07610-y (PMC13760791; doi:10.1186/s13071-026-07610-y)
Supplement: Supplementary file 1 [file 13071_2026_7610_MOESM1_ESM.docx]

**Supplementary file 1: Figure S1**. Photographs of sampling sites, snail counting, identification, and cercarial shedding techniques

**Fig. S1:** Physicochemical water quality testing (a, b), Snail scoping and handpicking (c, d), morphological snail identification (e), snails exposed to light in shedding wells (f, g), and cercarial identification under a dissecting microscope (h).
