# Supplementary material for: Spatio-ecological determinants of Biomphalaria and Bulinus snail intermediate hosts and schistosome-like infections in the Lango subregion, Northern Uganda: a geostatistical approach to guide targeted disease control
Source: Parasit Vectors. 2026 Aug 18;19:492. doi: 10.1186/s13071-026-07610-y (PMC13760791; doi:10.1186/s13071-026-07610-y)
Supplement: Supplementary file 2 [file 13071_2026_7610_MOESM2_ESM.docx]

**Supplementary file 2: Figure S2**. Photographs of intermediate freshwater snails collected and identified morphologically to species levels.

**Fig. S2:** Snail species a (*Biomphalaria choanomphala)*, b (*B. pfeifferi*), c (*B. sudanica)*, d (*Bulinus africanus)*, e (*B. nasutus),* f (*B. globosus)*, g (*B. ugandae)*, h (*Lymnaea natalensis)*, i (*Mellanoides tuberculate)*, (j) (*Pila ovata)* and k (*Bivalves) were identified*.
