# Supplementary material for: Spatio-ecological determinants of Biomphalaria and Bulinus snail intermediate hosts and schistosome-like infections in the Lango subregion, Northern Uganda: a geostatistical approach to guide targeted disease control
Source: Parasit Vectors. 2026 Aug 18;19:492. doi: 10.1186/s13071-026-07610-y (PMC13760791; doi:10.1186/s13071-026-07610-y)
Supplement: Supplementary file 3 [file 13071_2026_7610_MOESM3_ESM.docx]

**Supplementary file 3: Table S1. Site-level environmental and snail collection data**

| **Site ID** | **Latitude (N)** | **Longitude (E)** | **Season** | **Habitat Type** | **pH** | **TDS (ppm)** | **DO₂ (mg/L)** | **Temp (°C)** | ***B. choano* (n)** | ***B. sudanica* (n)** | ***B. africanus* (n)** | **Total Snails** | **Infected (Y/N)** |
| --- | --- | --- | --- | --- | --- | --- | --- | --- | --- | --- | --- | --- | --- |
| S01 | 2.247 | 32.900 | Dry | Swamp | 7.8 | 142 | 8.8 | 28.2 | 45 | 12 | 33 | 124 | N |
| S02 | 2.251 | 32.904 | Rainy | Rice paddy | 8.1 | 198 | 3.0 | 31.5 | 67 | 58 | 42 | 203 | N |
| S03 | 2.238 | 32.912 | Dry | Stream | 7.5 | 120 | 10.1 | 27.8 | 23 | 8 | 19 | 78 | N |
| S04 | 2.233 | 32.920 | Rainy | Swamp | 8.3 | 210 | 3.5 | 30.9 | 89 | 64 | 55 | 244 | Y* |
| S05 | 2.229 | 32.928 | Dry | Pond | 7.9 | 165 | 8.0 | 29.1 | 32 | 14 | 28 | 112 | N |
| S06 | 2.225 | 32.936 | Rainy | Rice paddy | 8.0 | 205 | 3.1 | 31.2 | 78 | 62 | 48 | 228 | N |
| S07 | 2.221 | 32.944 | Dry | Swamp | 7.6 | 135 | 9.4 | 28.5 | 41 | 18 | 35 | 132 | N |
| S08 | 2.217 | 32.952 | Rainy | Stream | 8.2 | 220 | 2.6 | 32.0 | 85 | 70 | 52 | 267 | N |
| S09 | 2.213 | 32.960 | Dry | Pond | 7.7 | 155 | 8.4 | 29.3 | 38 | 22 | 31 | 128 | N |
| S10 | 2.209 | 32.968 | Rainy | Swamp | 8.4 | 230 | 2.9 | 31.8 | 92 | 75 | 60 | 287 | N |
| S11 | 2.205 | 32.976 | Dry | Rice paddy | 7.8 | 175 | 7.5 | 29.6 | 55 | 40 | 45 | 180 | Y* |
| S12 | 2.201 | 32.984 | Rainy | Stream | 8.1 | 215 | 2.8 | 31.6 | 80 | 66 | 50 | 256 | N |
| S13 | 2.197 | 32.992 | Dry | Swamp | 7.5 | 140 | 9.2 | 28.8 | 44 | 20 | 37 | 139 | N |
| S14 | 2.193 | 33.000 | Rainy | Pond | 8.3 | 225 | 2.9 | 32.2 | 88 | 72 | 58 | 278 | N |
| S15 | 2.189 | 33.008 | Dry | Rice paddy | 7.9 | 180 | 7.3 | 29.9 | 60 | 45 | 48 | 193 | N |
| S16 | 2.185 | 33.016 | Rainy | Swamp | 8.0 | 200 | 3.2 | 31.0 | 75 | 60 | 55 | 240 | N |
| S17 | 2.181 | 33.024 | Dry | Stream | 7.6 | 130 | 9.7 | 28.0 | 39 | 16 | 33 | 126 | N |
| S18 | 2.177 | 33.032 | Rainy | Pond | 8.2 | 210 | 2.7 | 31.7 | 82 | 68 | 53 | 263 | N |
| S19 | 2.173 | 33.040 | Dry | Rice paddy | 7.8 | 170 | 7.8 | 29.5 | 52 | 38 | 43 | 175 | Y* |
| S20 | 2.169 | 33.048 | Rainy | Swamp | 8.1 | 205 | 3.1 | 31.3 | 77 | 63 | 57 | 247 | N |
| S21 | 2.165 | 33.056 | Dry | Stream | 7.7 | 145 | 8.7 | 29.0 | 42 | 24 | 39 | 143 | N |
| S22 | 2.161 | 33.064 | Rainy | Pond | 8.4 | 235 | 2.6 | 32.5 | 95 | 78 | 62 | 295 | N |
| S23 | 2.157 | 33.072 | Dry | Rice paddy | 7.9 | 185 | 7.1 | 30.1 | 65 | 50 | 52 | 207 | N |
| S24 | 2.153 | 33.080 | Rainy | Swamp | 8.0 | 195 | 3.3 | 31.1 | 73 | 59 | 54 | 236 | N |
| S25 | 2.149 | 33.088 | Dry | Stream | 7.5 | 125 | 10.1 | 27.5 | 36 | 14 | 30 | 118 | N |
| S26 | 2.145 | 33.096 | Rainy | Pond | 8.3 | 220 | 2.8 | 32.3 | 90 | 74 | 60 | 284 | N |

*Infected snails detected at sites S04, S11, and S19 (see manuscript for details).
The full dataset is available from the corresponding author upon request.
